# The Surgery of Fractures

**Published:** 1916-05-13

**Authors:** 


### PEACE AND WAR.

### The Surgery of Fractures.

Within the present century there have been introduced into surgical science several very important innovations in the treatment of fractured bones; some involving novel, extensive, and difficult operative procedures, some the use of remedial agents previously neglected, some the improvement and extension of the principles of treatment which up to the end of the nineteenth century had been unchallenged and accepted dogmata. As an instance of the first category, no more striking example can be quoted than the operative measures first proposed and practised by Sir W. Arbuthnot Lane; of the second category the teachings of Lucas-Championnière about mobilisation and massage are the type; and in the third a multitude of splints and other devices, of all materials, testify to the ingenuity of surgeons and instrument-makers.

Conservatism in Fracture Treatment.

Notwithstanding these advances, and much experimental work concerning the growth and repair of bone done upon animals, it is undeniably the fact that large sections of the medical profession rely in the treatment of fractures upon precisely those methods and contrivances which were in use before the development of $x$-ray technique and the progress of aseptic surgery had led the pioneer workers to modify ancient practice. To put it baldly, the profession as a whole has not been sufficiently impressed by the newer methods to "scrap" the old-fashioned ways of treating fractures. This may conceivably be due to various reasons: such as pig-headed stupidity on the part of the medical profession, failure of the advocates of a new method to convince their colleagues of its superiority to older plans (owing either to poor advocacy or to poor results), or confusion arising through the conflicting and contradictory claims of innovators upholding different principles and methods. Possibly all three of these factors have had something to do with the conservative tendencies just mentioned. There has been, we believe, a certain reluctance on the part of the rank and file of the profession to keep up with the times; though this is probably the least important of the factors at work, for, in spite of the contrary opinion entertained by some specialists (of whom Mr. Groves appears to be one), the bulk of the medical profession is not sunk in idiotic and obstinate lethargy. Certainly the occasional failures, and disastrous failures at that, of revolutionary methods and procedures have had something to do with the matter. But in the writer's opinion the third alternative is as much responsible as any for the partial failure of the medical profession to keep abreast of scientific advance in the treatment of fractures—namely, the conflicting advice of those who uphold new methods indeed, but not the same methods. One teacher is eloquent on behalf of mobilisation and massage for almost every case of fracture; another extols the merits of screws and plates for cases of an identical nature; a third explains that the use of his newly devised method of securing extension is all that is needed; another sticks closely to last century's methods and inculcates them on his students; and confusion, naturally enough, results.

It is the outstanding merit of the latest addition to the literature of fractures * that all methods are passed under review, and the indications for each sanely and impartially expounded. Mobilisation and massage, for instance, are clearly explained, and their value is recognised: the cases in which they are suitable, to the exclusion of all other methods, are indicated; a preliminary trial before adopting more radical measures is recommended in many types of fracture, and the limitations by which to judge whether such a trial shall be continued or abandoned are laid down. As a side issue, of small intrinsic importance, it is just worth noting that Mr. Groves, like many other surgeons, entirely ignores the work of Sir W. H. Bennett in teaching and practising the Championnière methods years and years before the younger surgeon whose advocacy of massage for fractures has lately brought him into prominence. Treatment by mechanical methods, especially by splints, combined with extension, is also fully discussed; and operative measures are proposed only for those cases where the prospect of a satisfactory result by less stringent means is deemed to be not properly assured.

The Relation of Structure and Function.

Opinions may differ, of course, as to what constitutes a "satisfactory result" in a case of fracture. But since the $x$-rays provided such a wealth of information as to the anatomical results obtained in the repair of fractures, the correlation of functional utility with exact anatomical repair has been worked out in a way which was never possible before; and Mr. Groves has taken a share in this very necessary attempt to found firmly the basic principles and aims of treatment. It is well enough known to every practitioner that many persons recover perfect use of a broken limb, even though gross deformity thereof persist; and that a perfect restoration of form is not invariably accompanied by a good functional result. These facts have occasionally obscured the vast importance of securing the best possible anatomical restoration of a broken bone as the best means of ensuring the utility of the limb. Mr. Groves exhibits the facts, as disclosed by the researches of the Fracture Committee of the British Medical Association, in a telling antithesis: in a hundred cases with good form (in the repaired bone) there are ninety with good function; in a hundred cases with bad form there are thirty with good function. Obviously, then, the securing of a good anatomical result is one of the main desiderata. One or other of the now numerous varieties of operative treatment is frequently necessary for this purpose; and a large part of the book is accordingly given up to the principles and

* *On Modern Methods of Treating Fractures.* By E. W. H. Groves, M.D., F.R.C.S. Bristol: J. Wright and Sons, 1916. Pp. 286.

practice of this comparatively new science. In this department Mr. Groves is an expert, and his advice is eminently sound and trustworthy. Every hospital surgeon and house surgeon should study his pages carefully, for they contain the latest ideas and the most ingenious details of their application.

FRACTURES DUE TO GUNSHOT WOUND.

In military surgery the treatment of wounds involving fracture of bones is one of the most complicated and difficult problems which our surgeons have to solve. On this subject Major Groves has some excellent advice, very emphatically and plainly expressed. He denounces the treatment of such fractures by screws and plates as utterly unjustified, either by theory or practice; and outlines a scheme of improved splint with extension treatment which he considers far preferable. This chapter well deserves the study of all those surgeons who have the care of wounded soldiers at home or abroad, and may act as a warning to some of them who have let enthusiasm outrun discretion. Major Groves' book is a valuable addition to surgical literature and a real credit to British surgery.

